# Dogs can detect an odor profile associated with *Staphylococcus aureus* biofilms in cultures and biological samples

**DOI:** 10.3389/falgy.2024.1275397

**Published:** 2024-02-13

**Authors:** Meghan T. Ramos, Gerard Chang, Clara Wilson, Jessica Gilbertie, James Krieg, Javad Parvizi, Antonia F. Chen, Cynthia M. Otto, Thomas P. Schaer

**Affiliations:** ^1^ Penn Vet Working Dog Center, Clinical Sciences and Advanced Medicine, School of Veterinary Medicine, University of Pennsylvania, Philadelphia, PA, United States; ^2^ Department of Orthopaedics, Geisel School of Medicine at Dartmouth, Lebanon, NH, United States; ^3^ Center for One Health Research Edward Via College of Osteopathic Medicine, Blacksburg, VA, United States; ^4^ Rothman Orthopaedic Institute, Philadelphia, PA, United States; ^5^ Department of Orthopaedics, Harvard Medical School, Brigham and Women's Hospital, Harvard University, Boston, MA, United States; ^6^ Department of Clinical Studies New Bolton Center, School of Veterinary Medicine, University of Pennsylvania, Kennett Square, PA, United States

**Keywords:** canine detection, *Staphylococcus aureus*, biofilm, volatile organic compound (VOC), olfaction, periprosthetic joint infection (PJI), culture-negative infection

## Abstract

**Introduction:**

The study investigated the utilization of odor detection dogs to identify the odor profile of *Staphylococcus aureus (S. aureus)* biofilms in pure *in vitro* samples and in *in vivo* biosamples from animals and humans with *S. aureus* periprosthetic joint infection (PJI). Biofilms form when bacterial communities aggregate on orthopedic implants leading to recalcitrant infections that are difficult to treat. Identifying PJI biofilm infections is challenging, and traditional microbiological cultures may yield negative results even in the presence of clinical signs.

**Methods:**

Dogs were trained on pure *in vitro S. aureus* biofilms and tested on lacrimal fluid samples from an *in vivo* animal model (rabbits) and human patients with confirmed *S. aureus* PJI.

**Results:**

The results demonstrated that dogs achieved a high degree of sensitivity and specificity in detecting the odor profile associated with *S. aureus* biofilms in rabbit samples. Preliminary results suggest that dogs can recognize *S. aureus* volatile organic compounds (VOCs) in human lacrimal fluid samples.

**Discussion:**

Training odor detection dogs on *in vitro S. aureus*, may provide an alternative to obtaining clinical samples for training and mitigates biosecurity hazards. The findings hold promise for culture-independent diagnostics, enabling early disease detection, and improved antimicrobial stewardship. In conclusion, this research demonstrates that dogs trained on *in vitro S. aureus* samples can identify the consistent VOC profile of PJI *S. aureus* biofilm infections. The study opens avenues for further investigations into a retained VOC profile of *S. aureus* biofilm infection. These advancements could revolutionize infectious disease diagnosis and treatment, leading to better patient outcomes and addressing the global challenge of antimicrobial resistance.

## 1 Introduction

Each year in the United States, an estimated 750,000 people undergo hip or knee arthroplasty. According to the National Joint Registry, up to 12% of the hip and 22% of the knee arthroplasties will require a revision surgery due to a periprosthetic joint infection (PJI) (1). PJIs are the leading cause of failure due to their ability to form bacterial biofilms in or around the orthopedic implants (1–3). Biofilms are microbe-derived sessile communities attached to orthopedic implants (1, 4, 5). Once attached, the bacterial biofilms produce a barrier of extracellular polymeric substances (EPS) known as a “slime layer”. The magnitude of virulence is dictated by the specific characteristics of the causative agent(s) (6–8). *Staphylococcus aureus* (*S. aureus*) is the most common biofilm in PJI (1, 3, 4, 9). *S. aureus* virulence is demonstrated in the evasion of the immune system, antibiotic resistance, and ability to disperse via detachment of planktonic bacteria (4, 10).

Identifying the causative organism(s) in musculoskeletal infection, such as fracture related injections (FRI) or PJI, is a challenging task and recent work continues to demonstrate the shortcomings of traditional microbiological cultures. Approximately 20%–50% of musculoskeletal infection patients have negative cultures despite clinical signs and laboratory evidence being consistent with PJI or FRI (11). These findings have produced a new clinical entity designated to be “culture-negative infections”. Moreover, biofilm research has intensified and with the growing burden of biofilm infection, clinical microbiology has undergone a paradigm shift from traditional culture-based to molecular-based methods (12–14). The clinical dilemma of the culture-negative patient is not limited to PJI or FRI, but is a common clinical entity across many areas of medicine (15). Consequently, infectious disease physicians are confronted with empirical treatment choices often requiring multiple courses of broad spectrum antimicrobial regimes when treating culture negative patients. These practices continue to compromise antimicrobial stewardship guidelines amplifying the global threat of antimicrobial resistance (16).

Despite considerable efforts to overcome the diagnostic challenges related to biofilm infections and culture negative results by maximizing microbiological culture yield, the sensitivity of culture still ranges from 39%–70% (11). This comes as no surprise since it is now accepted, following the arrival of polymerase chain reaction (PCR), that <2% of all pathogens that exist are culturable (17). There is convincing data from other medical fields that molecular methods enhance the detection of pathogens missed by traditional cultures and can lead to favorable clinical outcomes with targeted antimicrobial therapy (18–20). In the context of PJI and FRI, the rapidly evolving field of molecular techniques for infectious disease diagnosis will undoubtedly have an important clinical implication. While PCR is a promising tool in the detection of biofilm infections, research demonstrates PCR is not superior to culture (18). As such, it is important to investigate emerging and potentially complementary methods for biofilm detection.

Recently, harnessing the field of molecular techniques, an area of interest has emerged: characterizing specific volatile organic compound (VOC) profiles associated with disease. VOCs are organic chemicals produced as byproducts of metabolism in all living organisms, from bacteria to humans (21, 22). As potential biomarkers, unique VOC signatures have been associated with numerous infectious, metabolic, and neoplastic diseases (21–24). The VOCs associated with disease may originate from altered metabolism of the host, the metabolism of the etiologic agent itself or an interaction between the two (25). These VOCs can be detected in a wide array of biosamples, such as blood, sweat, lacrimal fluid (fluid secreted by the lacrimal glands to lubricate the eye), breath, urine, feces, and tissue. The VOC profile from a single sample can be assessed using chemosensors (e.g., gas chromatography-mass-spectroscopy), electrosensors (e.g., DNA coated nanosensors) and biosensors (e.g., dogs, rats) (23, 26–28).

The focus of this study is utilizing trained dogs to provide proof-of-concept information as to whether *S. aureus* biofilms have a detectable similar odor profile across both *in vitro* samples, derived from cultured *S. aureus*, and *in vivo* biosamples from animals with experimental infections and humans with spontaneous infections*.* VOCs may be used as culture-independent biomarker profiles to identify the infection etiology based on a known volatile metabolome profile for a specific infection. To that effect, this method may eliminate the uncertainty of successful microbial growth and the lengthy time required for generating pure diagnostic cultures. The detection of the *S. aureus* biofilm profile may also be achieved through a canine biosensor. Dogs have been shown to be effective disease biosensors due to their remarkable olfactory sensitivity in identifying, and specificity in discriminating, VOC signatures (26, 28–31). For example, dogs have been trained to detect VOCs associated with a wide range of diseases, including both noninfectious diseases such as cancer, and infectious diseases caused by bacteria, viruses, and prions (30–35). Compared to currently available chemosensors, dogs are capable of rapidly identifying VOC signatures amongst a complex array of volatiles which may lead to the potential to identify diseases at early stages when treatment options may be more effective (28). Dogs may additionally compliment approaches of analytical-organic chemistry, and “e-noses”, as has been demonstrated previously where dogs were trained to detect human ovarian cancer from blood plasma samples, alongside single stranded DNA-coated carbon nanotube sensors (36). However, an obstacle to the wide use of disease detection dogs lies in the reliance on a large number of samples from affected individuals and appropriate controls, which can pose challenges in achieving adequate sample diversity and generalization of subtle odors across a “noisy” background (37). One approach to simplify the training could be to train the dogs on the pure source of the VOCs [e.g., cultured tumor cells (38) or cultured pathogens (30, 31, 39)]. However, it is not currently known whether cultures suitably represent biosamples in practice. In the case of *S. aureus,* it is important to establish whether dogs can detect an odor associated with *S. aureus* (indicating that an odor profile exists), and whether dogs trained on cultured samples can generalize this information to non-human animal or human derived biosamples where the complexity of host-microbe interactions are well established. To achieve this, it is beneficial to create a well-controlled experimental test system using non-human animal models as a source of biosamples, so that the strain and volume of *S. aureus* biofilm can be monitored. Furthermore, the number of colony forming units (CFU (indicative of the bioburden of *S. aureus* bacteria present in the sample) is of interest as a specific threshold of recognition by the dog may fluctuate based on the number of *S. aureus* CFU emanating the VOC profile. Zhou et al. reported that only a subset (25%–34%) of *in vitro* VOCs reliably translate to *in vivo* detection, and their animal model studies have shown that breath VOCs can be used to identify infection etiology, even down to the strain level for the bacterial pathogen (40). Alongside questions regarding the level of infection required for recognition, it is of interest and clinical relevance to explore if dogs trained on *in vitro S. aureus* biofilm can continue to detect this VOC profile in experimental *S. aureus* biofilm infections and in clinical patients of confirmed *S. aureus* infections with PJI or FRI.

As such, this study sought to answer three research questions to establish whether dogs can identify an odor profile associated with *S. aureus* biofilms in *in vivo* biosamples (lacrimal fluid) and to assess the utility of *S. aureus* cultures as a training aid. The research questions are: (1) can dogs trained on *in vitro S. aureus* (10^6^ CFU – moderate infection load) identify biosamples (lacrimal fluid) taken from an *in vivo* animal model (rabbit) infected with moderate *S. aureus* biofilm, (2) can dogs trained on *in vitro* S. *aureus* (10^4^ CFU – mild infection load) identify biosamples (lacrimal fluid) taken from an *in vivo* animal model (rabbit) infected with mild *S. aureus* biofilm, and (3) can the dogs involved in research questions 1 and 2 identify *S. aureus* in biosamples (lacrimal fluid) taken from humans with confirmed *S. aureus* PJI.

## 2 Materials and methods

### 2.1 Sample types

#### 2.1.1 *In vitro* training samples

2.1.1.1

Moderate bacterial load *in vitro S. aureus* samples were obtained from *S. aureus* (10^6^ CFU) cultured on 316l stainless steel chips from orthopedic fracture plates and incubated for 16 h at 37°C in Mueller Hinton Broth (MHB) in a 50 ml culture tube. Following the incubation period, each batch of samples was separately cultured to ensure no contamination occurred during the incubation process. The cotton plugs from the headspace of each culture tube (Fisherbrand™) were aseptically cut into smaller sample sizes (1/2, 1/4, 1/8, 1/16, 1/32) of the original cotton plug, transferred into sterile glass jars with lids, and stored at −80°C until use.

2.1.1.2

Mild *in vitro S. aureus* samples were obtained by placing a cotton plug in the headspace of *S. aureus* (10^4^ CFU) cultured on 316l stainless steel chips from orthopedic fracture plates and incubated for 16 h at 37°C in Mueller Hinton Broth (MHB) in a 50 ml culture tube and processed using the same methods as for *S. aureus* (10^6^ CFU).

2.1.1.3

Control *in vitro* VOC samples were obtained by placing a cotton plug in the headspace of 50 ml sterile culture tubes containing 316l stainless steel chips from orthopedic fracture plates and incubated for 16 h at 37°C in Mueller Hinton Broth (MHB). Following the incubation period, a small portion of each batch of samples were re-cultured to ensure no contamination occurred during the incubation process. Other controls/distractors in this stage: fresh cotton plugs that were not exposed to bacteria or media, unused Schirmer tear strips (Merck & Co., Inc., Rahway, NJ), laboratory nitrile gloves, and isopropyl alcohol (2 ml), were placed in the glass jars used for odor presentation.

#### 2.1.2 *In vivo* testing samples

##### 2.1.2.1 *In vivo S. aureus* lacrimal fluid samples from rabbits in cohorts 1 and 2

As part of an IACUC (protocol #805734) approved study, a periprosthetic joint infection model was induced in the right stifle joints of skeletally mature New Zealand White male castrated and female rabbits (*n* = 24 cohort 1, *n* = 16 cohort 2; 50:50 ratio male castrated vs. female). The rabbits were housed in stainless steel cages having auditory, olfactory, and visual contact with each other. Their diet consisted of a pelleted diet and a small amount of fresh roughage, with free access to hay and water. Animals were housed in a room with a 12:12 h light cycle and controlled temperature (70.82 ± 0.1 °F) and humidity (35.9% ± 6.2%). All animals were monitored daily by a veterinarian from the principal investigator's laboratory throughout the entire study.

Anesthesia: On the day of surgery, rabbits fasted for 12 h, were induced with an intramuscular injection of ketamine (35 mg/kg) (Ketamine HCl, Covetrus) and xylazine (2.5 mg/kg) (Xylazine Injection, Covetrus). Propofol (PropoFloTM 28, Zoetis) was administered as intravenous bolus (0.5–1 mg/kg) if anesthetic depth was not sufficient to perform the intubation procedure. Glycopyrrolate (0.01 mg/kg) (Glycopyrrolate injection, BluePoint) was administered intravenously if bradycardia (defined as heart rate less than 120 beats per minute) with concurrent hypotension (defined as mean arterial pressure less than 60 mmHg) occurred. Approximately ten minutes after premedication, the right or left auricular vein and artery of the same ear were catheterized with a 24 Ga (artery) and a 22 Ga catheter (vein) (Surflo, Terumo) respectively. Then a v-gel® (v-gel®, Docsinnovent), was placed and confirmed by capnography, a lubricated 3.5 French (∼1.2 mm external diameter), 40 cm polypropylene catheter (Sovereign Urinary Catheter, Henry Schein Animal Health) was inserted through the v-gel® airway channel. The v-gel® was then removed and a 3 mm internal diameter (ID) cuffed ETT (Sheridan/CF®, Teleflex Medical) was threaded over the catheter. After insertion of the ETT, the polypropylene catheter was removed. The cuff was inflated to 20 mmHg (19) measured with an aneroid manometer connected via a three-way stopcock to the cuff balloon. After intubation, the animals were connected to a circle breathing system designed for animals under 7 kg body weight (Anesthesia WorkStation, Hallowell EMC) and maintained on inhalation anesthesia using 1%–5% Isoflurane in oxygen delivered through a circle breathing circuit at 1l/min. Fraction of inspired oxygen (FiO2), respiratory rate (RR) and end tidal concentration of CO2 (ET CO2) were recorded every five minutes. FiO2 and end tidal CO2 were measured with sidestream capnography and gas analysis. Pulse oximetry, ECG, invasive blood pressure (IBP), FiO2, end-tidal carbon dioxide (ETCO2) and respiratory rate (RR) were recorded throughout each anesthetic event using a multiparameter monitor (Datex Ohmeda S/5, GE). Rectal temperatures were recorded throughout the anesthetic session. The rabbits were maintained in dorsal recumbency with the forelimbs and hind limbs extended and head aligned with the spine. Topical 2% lidocaine (0.2 ml) (Lidocaine 2%, VetOne) was applied via atomizer to the larynx two minutes prior to intubation. The v-gel® was lubricated with lidocaine gel (Lidocaine Hydrochloride Oral Topical Solution 2%, Akorn) mixed with regular sterile lubricant (VetOne® OB Lube), and the ETT was lubricated (VetOne® OB Lube).

Surgery: With animals is left lateral recumbency, the right stifle region was clipped, aseptically prepped, and draped. A ∼1.5 cm–2 cm arthrotomy incision was made medial and in parallel to the middle patellar ligament. Synovial fluid was sampled. The fossa intercondylaris was identified, and a 2 mm K-wire was used to open the femoral canal. The PJI model utilized a 3.5 mm cortex stainless-steel screw in combination with an ultra-high molecular weight polyethylene (UHMWPE) washer placed in the intercondylar fossa of the distal femur extending 1/3 into the medullary canal of the femur. The joint capsule and subcutaneous tissues were closed using resorbable sutures. The skin was closed using a subcuticular pattern followed by surgical glue. Ten days later, under the same general anesthesia protocol, the right femorotibial joint was samples for synovial fluid and inoculated with 0.1 ml saline containing 1 × 10^6^ CFU *S. aureus* for cohort 1 and 1 × 10^4^ CFU *S. aureus* for cohort 2. No systemic antimicrobials were given at any time. Thirty-eight days after inoculation the rabbits were sacrificed with an overdose of a commercially available euthanasia solution (Pentobarbital 1 ml/5 kg) according to the guidelines set forth by the current AVMA Panel on Euthanasia and ex vivo analyses consisted of bacteriology, imaging of the implant surface topography using confocal microscopy and scanning electron microscopy for confirmation of *S. aureus* biofilm. Lacrimal fluid was collected preoperative for one week, on the day of surgery and every day postoperative for the duration of the study from both the right and left eye using a sterile Schirmer tear strip (Merck & Co., Inc., Rahway, NJ) using mild physical restraint throughout the duration of the study by one handler while a 2nd handler performed the tear film collection. The tear strip was placed in the medial canthus of each eye for 1 min and then immediately transferred to non-evacuated blood tubes and stored at −80°C until use.

##### 2.1.2.2 Control lacrimal fluid samples from rabbits in cohorts 1 and 2

Lacrimal fluid collected preoperatively for one week from both the right and left eyes using a Schirmer tear strip. The tear strip was placed in the medial canthus of each eye for 1 min and then transferred to non-evacuated blood tubes and stored at −80°C until use. These samples were used as uninfected biologic controls for the *in vivo* testing for research questions one and two of the study. Background controls for this stage were unused sterile Schirmer tear strips. Other controls/distractors in this stage: fresh cotton plugs that were not exposed to bacteria or media, laboratory nitrile gloves, and isopropyl alcohol (2 ml), were placed in the glass jars used for odor presentation. A summary of samples used in this stage can be seen in Table 1.

**Table 1:** Summary of sequential stages of training and testing.

Training				
Stage	Target Sample	Background control	Distractor control	Equipment
Stage 1	Pure *in vitro S. aureus*	Cotton plug		Odor jars only
Stage 2	Pure *in vitro S. aureus*	Cotton plug, MHB broth incubated cotton plug.	Isopropyl alcohol, Schirmer tear strips, nitrile gloves	12- port odor
Stage 3	Pure *in vitro S. aureus*	Cotton plug, MHB broth incubated cotton plug	Isopropyl alcohol, Schirmer Tear Strips, control lacrimal samples (rabbits)	8- port odor wheel
Stage 4	Pure *in vitro S. aureus*	Cotton plug, MHB broth incubated cotton plug	Isopropyl alcohol, Schirmer Tear Strips, control lacrimal samples (rabbits)	8 port odor wheel with target odor and blank odor wheels
Testing				
	Target Sample	Biologic controls	Background controls	Equipment
Research Question 1	*In vivo r*abbit lacrimal fluid biosample, *S. aureus* PJI cohort 1	Un-infected lacrimal samples (rabbits)	Schirmer tear strips	8 port odor wheel with target odor and blank odor wheels
Research Question 2	*In vivo r*abbit lacrimal fluid biosample, *S. aureus* PJI cohort 2	Un-infected lacrimal samples (rabbits)	Schirmer tear strips	8 port odor wheel with target odor and blank odor wheels
Research Question 3	*S. aureus* lacrimal fluid from human PJI patients	Lacrimal samples (humans)	Schirmer tear strips	8 port odor wheel with target odor and blank odor wheels

This table highlights the target, biologic controls, background controls, distractor control odors, and equipment used at each stage of the study.

MHB, mueller hinton broth.

#### 2.1.3 Human biosamples (lacrimal fluid)

##### 2.1.3.1 *S. aureus* lacrimal fluid from human PJI patients

Human biosamples (lacrimal fluid) from patients presenting to the Rothman Institute at Thomas Jefferson University, Philadelphia with a confirmed microbiological culture of *S. aureus* positive orthopedic implant infections were obtained. Participants were able to approve via standard of care consent form and were informed of the study aims and methods and were reminded of their right to withdraw from the study at any time (IRB #16D.634). Lacrimal fluid samples were obtained via sterile Schirmer tear strips placed in the medial canthus of each eye prior to general anesthesia and revision surgery. Samples were processed using the Guidance Regarding Methods for Deidentification of Protected Health Information in Accordance with the Health Insurance Portability and Accountability Act (HIPAA) Privacy Rule and immediately stored at −80°C until use.

#### 2.1.4 Control lacrimal fluid from human patients

Human lacrimal fluid from patients presenting to the Rothman Institute at Thomas Jefferson University, Philadelphia for an elective joint surgical procedure such as Total Knee Arthroplasty (TKA) or Total Hip Arthroplasty (THA). Participants were able to approve via standard of care consent form and were informed of the study aims and methods and were reminded of their right to withdraw from the study at any time. Lacrimal fluid samples were obtained via sterile Schirmer tear strips placed in the medial canthus of each eye prior to surgery. Samples were processed using the Guidance Regarding Methods for De-identification of Protected Health Information in Accordance with the HIPAA Privacy Rule and immediately stored at −80°C until use.

### 2.2 Odor detection dogs

Following Institutional Animal Care and Use Approval (protocols #805676 and 806052) and informed client consent, four Penn Vet Working Dog Center (PVWDC) dogs and four privately owned dogs were enrolled in this study. Dogs varied in breed and sex and ranged in age from 1 to 5 years old (Mean = 3.0). Prior to this study, all PVWDC dogs were trained on a non-biological training odor: Universal Detection Calibrant (UDC) (39) and all privately owned dogs had participated in the American Kennel Club Scent-Work™ odor detection sport or similar organized odor detection training. Information on the individual dogs is described in Table 2.

**Table 2:** Odor detection dog information including trained final response and stage of odor detection training.

Odor detection dog	Breed	Age (years)	Sex (Male/Female)		Trained final response	Reward type	Odor detection Participation	
							Training stage	Testing stage
Osa	German Shepherd	2.5	Female	PVWDC, UDC	Silent bark	Tug toy	4	1,2
Mizu	German Shepherd	1.5	Female	PVWDC, UDC	“Play” bow	Tug toy	4	2
Jake	Labrador Retriever	2.5	Male	PVWDC, UDC	Sit	Tennis ball	4	1,2
Zoe	Dutch Shepherd	2	Female	PVWDC, UDC	Sit	Food	4	1
Lily	Golden Retriever	4	Female	Private, AKC ScentWork™	Sit	Food	4	2,3
Bonny	Border Collie	5	Female	Private, AKC ScentWork™	Sit	Food	4	2,3
Bea	Doberman Pinscher	2.5	Female	Private, AKC Scent-Work™	Down	Food	4	2,3
Boltz	Dachshund	4	Male	Private, AKC ScentWork™	Down	Food	3	N/A

PVWDC, penn vet working dog center; AKC, American kennel club; UDC, universal detection calibrant.

### 2.3 Training of odor detection dogs with *in vitro S. aureus* samples

#### 2.3.1 Training stages

##### 2.3.1.1 Stage 1 of *in vitro S. aureus*

The operant odor discrimination training involved pairing the odor of interest (cotton incubated with cultured *S. aureus*) with a trained final response, such as sit or down (see Table 1). Positive reinforcement in the form of a reward, such as a dog treat or toy, was used to reinforce the correct final response. Trained final response behavior to known negative samples was not rewarded. Table 2 highlights the training stages used in this study. The initial training began by exposing the dogs to the whole cotton plug incubated with *S. aureus* contained in a glass odor jar and rewarding the dog for sniffing the sample, establishing a positive association between the odor of interest and the reward. The dogs were trained to perform a trained final response upon identifying the target sample. The final responses were reinforced using a conditioned secondary reinforcer (“clicker”) to mark the correct response. This training was conducted in one morning and one evening session (within the same day), with each dog receiving 10 odor exposures per session. Once the dogs exhibited the trained final response within five seconds of sniffing the target sample 90% of the time in two consecutive training sessions the dogs were moved to training stage 2.

##### 2.3.1.2 Stage 2 of *in vitro S. aureus*

Stage 2 of training introduced the use of an odor wheel. The protocol for sample placement within the odor wheel consisted of a randomized assignment of target (cultured *S. aureus*), background controls, and control/distractor odor locations with each port designated by its' number space on the wheel (https://www.randomizer.org/). Each trial was documented on a written record and video recorded on a mounted tripod for review of the dogs' behavior at each port. Nitrile gloves were used and changed between handling of samples and whenever interacting with the odor wheel. The ports of the wheel were wiped with 70% isopropyl alcohol between each trial, the entire wheel was wiped with 70% isopropyl alcohol between each dog, and the floor beneath the wheel was cleaned with 70% isopropyl alcohol each day. A trial was defined as each time the dog was sent to search the wheel. A session was defined as all the consecutive trials completed by the dog in one training or testing session. Training sessions contained ten trials and did not occur more than twice per day. A dog's response at each port was scored as a “1” for a negative response, indicating a dog sniffed a port and moved on, a “2” for a positive response, indicating the dog sniffed a port and gave their trained final response, or a “3” for a hesitation, indicating a change in behavior without the dog demonstrating their trained final response. Stage 2 of training utilized a 12-port odor wheel. The 12-port odor wheel was used in this stage to introduce the background controls and the control/distractor samples for odor discrimination between these samples and the target odor. Between each trial, the 12-port carousel was rotated so that the location of the target sample was moved whereas the 8-port wheel described below allowed the samples to be removed and replaced to create a novel order of samples for each trial. Once the dogs exhibited the trained final response at the target sample with a 90% sensitivity and at the controls with a 95% specificity in three consecutive training sessions the dogs were moved to training stage 3.

##### 2.3.1.3 Stage 3 of *in vitro S. aureus*

The dogs were transitioned to a newly acquired odor wheel with 8-ports in training stage 3. This wheel was utilized as it had some advantages over the 12-port wheel, namely, the samples could be moved independently (allowing the order of samples presented to be changed each trial), and additionally had stainless-steel barriers between each port, aiding in keeping the odor cones from each sample separated. The dogs were trained to search in a clockwise direction of the 8-port circular odor wheel, sniffing each port in order, correctly sniffing, and passing both the background and distractor control samples, and exhibiting their trained final response behavior when they detected the target odor: cultured *S. aureus*. Training sessions contained ten trials and did not occur more than twice per day. A dog's response at each port was scored as a “1” for a negative response, indicating a dog sniffed a port and moved on, a “2” for a positive response, indicating the dog sniffed a port and gave their trained final response, or a “3” for a hesitation, indicating a change in behavior without the dog demonstrating their trained final response. For training stage 3, the same randomized sample placement described above was used to determine the target sample port location for each trial. Each port was designated by its' number space on the wheel (1–8) in the random generator. Once the dogs exhibited the trained final response at the target sample with 90% sensitivity and at the controls with 95% specificity in three consecutive training sessions the dogs were moved to training stage 4.

##### 2.3.1.4 Stage 4 of *in vitro S. aureus*

Training stage 4 introduced the dogs to the concept that the target odor may not be present in the wheel, where they were required to check and pass each port, performing a sit or down response on a low platform outside of the wheel space to indicate an “all clear” (referred to as a “blank wheel”). For training stage 4, the same randomized sample placement described above was used to determine the target sample port location for each trial or if the trial was a blank trial (no target odor). Each port was designated by its' number space on the wheel (1–8), a blank wheel was indicated as a number “9” in the random generator. Training stage 4 sessions contained ten trials once a day. A dog's response at each port was scored as a “1” for a negative response, indicating a dog sniffed a port and moved on, a “2” for a positive response, indicating the dog sniffed a port and gave their trained final response, or a “3” for a hesitation, indicating a change in behavior without the dog demonstrating their trained final response. Once the dogs reached 90% sensitivity and 95% specificity including blank wheels in three consecutive training sessions the dogs were moved to testing.

### 2.4 Testing for *S. aureus* profile of *in vivo* and human samples

#### 2.4.1 Testing protocols used for all research questions

**Figure 1:**
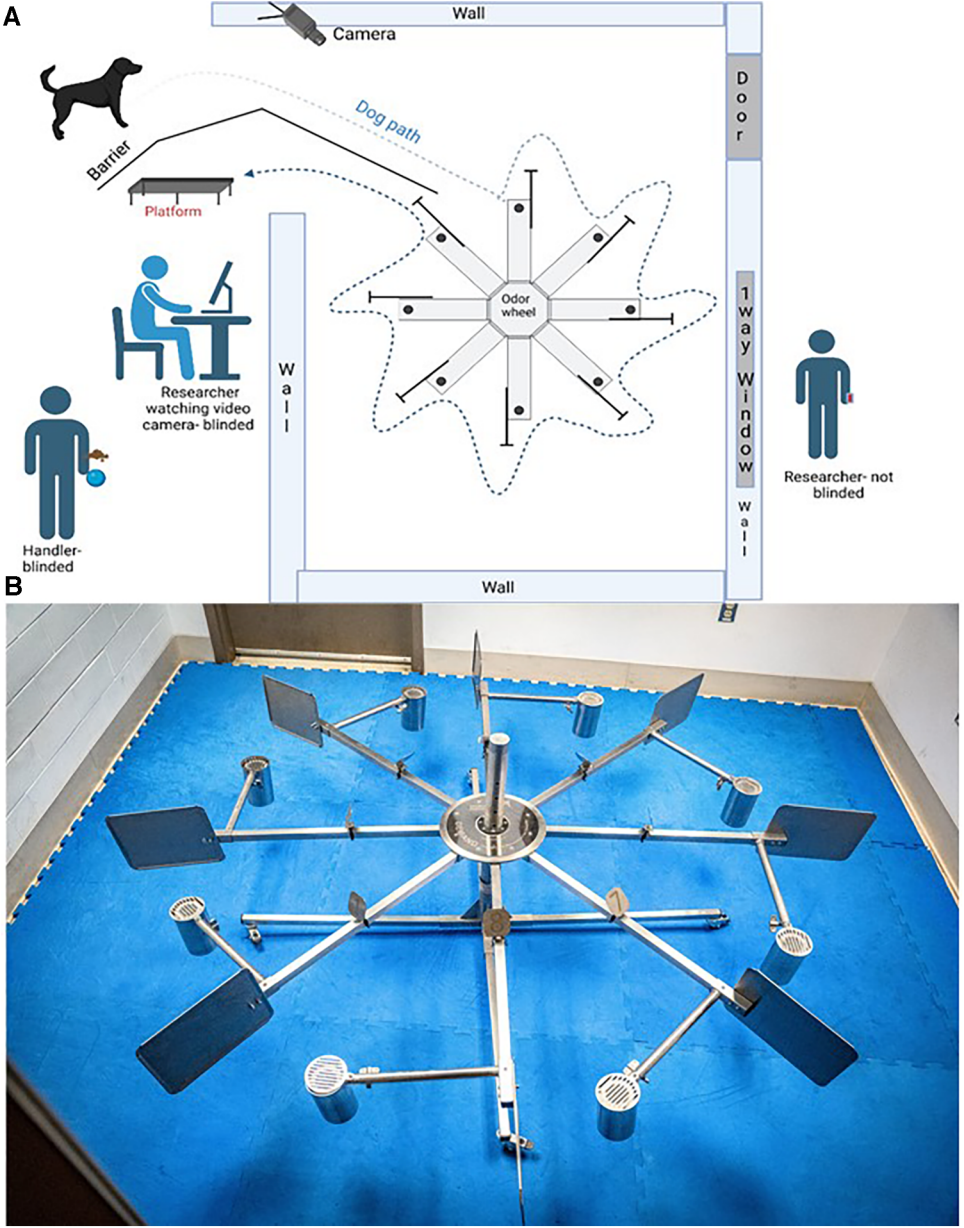
The laboratory setting for this study. (**A**) is the diagram of the laboratory set up. (**B**) is a photograph of the 8-port odor wheel.

The odor wheel searching pattern, cleaning, and randomization protocols used in training stages 3 and 4 were used for all testing stages. Only dogs that passed stage 4 of training proceeded to testing. Testing trials were performed once a day in a laboratory setting utilizing the 8-port odor wheel used during training (Figure 1). One dog was tested at a time and the order in which the dogs were to search during that session was randomized prior to the study day. Target, background and distractor control samples used for testing were removed from the 80°C storage twenty minutes prior to the testing session. Researchers changed gloves between handling of all samples thorough the session to avoid contamination. The odor wheel was located behind a barrier and monitored via a video feed so that the dogs could not see their handler, the recorder, or any observers during the testing trials. Testing trials were double blinded so that the dog and handler did not know where the target sample was placed on the wheel; however, the researcher who was out of the wheel room was aware. Because the handler did not know the location of the target, they were informed once the dog had made a response whether it was correct or incorrect by the researcher (out of sight from both the handler and dog) and the dog was either called away to reset for the next trial within the session (if incorrect) or, if correct, marked by a conditioned secondary reinforcer (a clicker) by the researcher and given a reward by the handler. Each testing session included 10 trials at least one of the trials was a blank wheel and a session was performed once per day. If a biologic control or background control was located in a port location beyond the target sample (example port 1) and the dog correctly alerted to the target sample, then the biologic and background controls were set to be presented in the blank wheel trial for the dog to evaluate the samples and provide a first pass response. First pass for each testing sample was recorded as a “1” for a negative response, indicating a dog sniffed a port and moved on (false negative), a “2” for a positive response, indicating the dog sniffed a port and gave their trained final response (true positive), and used for data analysis.

#### 2.4.2 Research question 1: can dogs trained on *in vitro S. aureus* (10^6^ CFU – moderate bacterial load) identify biosamples (lacrimal fluid) taken from an *in vivo* animal model (rabbit) infected with moderate *S. aureus* biofilm?

To address this research question, the cotton plug samples from the cultured *S. aureus* were replaced with the experimental PJI rabbit model biosamples (lacrimal fluid from infected rabbits). The biological controls were the lacrimal samples from uninfected rabbits. The background control samples for this phase consisted of a Schirmer tear strips. The distractor control odors were cotton plugs, MHB broth incubated cotton plug, isopropyl alcohol, and nitrile gloves (Table 2). The target samples for research questions two and three are highlighted below.

#### 2.4.3 Research question 2: can dogs trained on *in vitro S. aureus* (10^4^ CFU – mild bacterial load) identify biosamples (lacrimal fluid) taken from an *in vivo* animal model (rabbit) infected with mild *S. aureus* biofilm?

To answer this research question, the target samples were the biosamples (lacrimal fluid) obtained from the second cohort of infected rabbits which were inoculated with 1 × 10^4^ CFU *S. aureus*. The biological controls were the lacrimal samples from uninfected rabbits. The background control samples for this phase consisted of a Schirmer tear strips. The distractor control odors were cotton plugs, isopropyl alcohol, and nitrile gloves (Table 2).

#### 2.4.4 Research question 3: can the dogs involved in research questions 1 and 2 identify *S. aureus* in biosamples (lacrimal fluid) taken from human patients with confirmed *S. aureus* PJI?

To answer this research question, the target samples were the lacrimal fluid samples obtained from human patients presenting for revision surgery secondary to a confirmed *S. aureus* PJI. The biological controls included for this stage were control lacrimal samples (humans). These biological control lacrimal fluid samples were obtained preoperatively from patients undergoing routine orthopedic surgery. The background control samples of this stage were Schirmer tear strips. The distractor control samples were cotton plugs, isopropyl alcohol, Schirmer Tear Strips, and biological control lacrimal samples (rabbits),.

### 2.5 Statistical analysis

Sensitivity, specificity, positive predictive value, and negative predictive value were used to evaluate the performance of the odor detection dogs as a diagnostic test or screening tool for *S. aureus* positive and control samples. Sensitivity was determined as the true positives divided by true positives plus false negatives. Specificity was determined as specificity the number of true negatives divided by the number of true negatives plus the number of false positives. Positive predictive value (PPV) is defined as the true positives divided by true positives plus false positives and negative predictive value (NPV) as the number of true negatives divided by the number of true negatives plus the number of false negatives. A true positive was a final alert on the *S. aureus* target samples on the first pass of the port. A true negative was the correct omission of a final alert on the biological controls and the background control samples on the first pass of the ports. During the testing phase, only the dogs “first pass” response on the first presentation of the target *S. aureus* sample was considered for analysis. The probability of a correct trial by chance, where the dog exhibited their trained final response on the new target port and performed no false alert behaviors on the incorrect ports is 12.5%.

## 3 Results

### 3.1 Sample collection

#### 3.1.1 Training *in vitro S. aureus* samples

*S. aureus* samples were successfully cultured for this study without contamination of other bacteria as evidenced by the quality control cultures performed on each batch by the clinical microbiology laboratory at Penn Vet New Bolton Center following the incubation process.

#### 3.1.2 Testing *in vivo S. aureus* lacrimal fluid samples from rabbits

Biosamples obtained from the experimental PJI rabbits were obtained for this study are described in Table 3. In cohort 1, two rabbits developed sepsis and were euthanized and removed from the study on day 6 post inoculation. One of the rabbits in cohort 1 and cohort 2 developed ocular conjunctivitis prior the last sample collection and therefore only one sample was collected on day 7 and day 8 of those cohorts. Table 3 highlights the available samples for each day post inoculation. In cohort 1 and 2 a total of 43 and 47 positive *S. aureus lacrimal fluid* samples were obtained for screening by the dogs. 168 biological controls uninfected lacrimal fluid were collected in cohort 1 and 112 in cohort 2. For testing, the biological control sample was matched to the infected rabbit sample. The other biologic control samples were presented during blank wheel trials.

**Table 3:** The sensitivity, specificity, negative predictive value, and positive predictive value for research questions one and two.

**Research Question 1**: Can dogs trained on *in vitro S. aureus* (106 cfu – moderate bacterial load) identify biosamples (lacrimal fluid) taken from an *in vivo* animal model (rabbit) infected with moderate *S. aureus* biofilm?												
**Target sample:** *In vivo* rabbit lacrimal fluid *S. aureus* periprosthetic joint infection (PJI) cohort 1												
Days post inoculation with *S. aureus*	Positive Samples	First pass screenings (3 dogs)	Sensitivity* TP/(TP + FN)	Sensitivity %	Number of biologic and background controls presented	First pass controls screenings (3 dogs)	Specificity TN/(TN + FP)	Biologic and background Specificity %	NPV TN/(TN + FN)	NPV %	PPV TP/(TP + FP)	PPV %
Day 1	12	36	30/(30 + 6)	83%	12- matched rabbit biologic controls 24- Schirmer tear strips	108	102/(102 + 6)	94	102/(102 + 6)	94	30/ (30 + 6)	83
Day 3	12	36	32/(32 + 4)	88%	12- matched rabbit biologic controls 24- Schirmer tear strips	108	102/(102 + 6)	94	102/(102 + 4)	96	32/ (32 + 6)	84
Day 5	10	30	25/(25 + 5)	86%	10- matched rabbit biologic controls 20- Schirmer tear strips	90	87/(97 + 3)	96	87/(87 + 5)	94	25/ (25 + 3)	89
Day 7	9	27	25/(25 + 2)	93%	9- matched rabbit biologic controls 18- Schirmer tear strips	81	75/(75 + 6)	92	75/(75 + 2)	97	25/ (25 + 6)	80
**Research Question 2***:* can dogs trained on *in vitro S. aureus* (104 CFU – mild infection load) identify biosamples (lacrimal fluid) taken from an *in vivo* animal model (rabbit) infected with mild *S. aureus* biofilm, and 3)												
**Target sample:** *In vivo* rabbit lacrimal fluid *S. aureus* PJI cohort 2												
Days post inoculation with *S. aureus*	Positive Samples	First pass screenings (6 dogs)	Sensitivity* TP/(TP + FN)	Sensitivity %	Number of biologic and background controls presented	First pass control screenings (6 dogs)	Specificity TN/(TN + FP)	Biologic and background Specificity %	NPV % TN/(TN + FN)	NPV %	PPV % TP/(TP + FP)	PPV %
Day 2	12	72	63/(63 + 9)	87%	12- matched rabbit biologic controls 24- Schirmer tear strips	216	210/(210 + 6)	97%	210/(210 + 9) 96	96	63/(63 + 6)	91
Day 4	12	72	65/(65 + 7)	90%	12- matched rabbit biologic controls 24- Schirmer tear strips	216	201/(201 + 15)	93%	201/(201 + 7)	96	65/(65 + 15)	81
Day 6	12	72	56/(56 + 16)	77%	12- matched rabbit biologic controls 24- Schirmer tear strips	216	210/(210 + 6)	97%	210/(210 + 16) 93	93	56/(56 + 6)	80
Day 8	11	66	50/(50 + 16)	75%	11- matched rabbit biologic controls 22- Schirmer tear strips	198	187/(187 + 11)	94%	198/(198 + 16)	92	50/(50 + 11)	82

NPV, negative predictive value; PPV, positive predictive value.

* Specificity was calculated based on the biological controls and background controls; distractors were not included in this calculation.

#### 3.1.3 Testing lacrimal fluid samples from human patients

Eight control human lacrimal samples (left or right eye) were obtained from eight healthy patients. Three lacrimal fluid samples (left or right eye) were obtained from three PJI methicillin sensitive *Staphylococcus aureus* (MSSA) patients as confirmed by culture following surgical revision.

### 3.2 Detection dogs

#### 3.2.1 Detection dog training

Eight odor detection dogs were recruited for this study. Seven out of the eight dogs completed all four stages of training as outlined in Table 1. Boltz failed to complete training stage four due to lack of indication on the platform when searching the blank wheel. Boltz tried to re-search the wheel after reaching port 8 instead of indicating on the platform and was subsequently removed from further training or progressing to testing.

#### 3.2.2 Detection dog testing

Of the seven dogs, three participated in the first research question testing (Osa, Jake, and Zoe), six dogs participated in research question two testing (Osa, Mizu, Jake, Lily, Bonny, and Bea), and three dogs participated in research question three (Lily, Bonny, and Bea). Zoe was excluded after research question one because she was purchased for a career in law enforcement from the PVWDC program prior to the start of testing research question two. Mizu was trained to replace Zoe for testing research question two. Osa, Mizu, Jake, and Zoe were excluded from testing research question three because they were purchased for their working dog careers prior to the start of testing. For research questions one and two, analysis for differences between each dog and between dogs and specific rabbits were analyzed. There was no significant difference amongst dogs or towards a particular rabbit sample and therefore analysis of days post inoculation was investigated.

### 3.3 Research question one

The sensitivity and specificity of the three dogs (Osa, Jake, and Zoe) tested for research question one (target odor was the lacrimal fluid from mild *S. aureus* inoculated rabbits) are greater than 83% sensitivity and 92% specificity. The sensitivity improved as the days post-inoculation increased (days 1–7). PPV was greater than 80% and NPV was greater the 94%. Table 3 summarizes the sensitivity, specificity, negative predictive value, and positive predictive.

### 3.4 Research question two

The sensitivity and specificity of the six dogs (Osa, Mizu, Jake, Lily, Bonny, and Bea) tested for research question two (where the target odor was the lacrimal fluid from the rabbits inoculated with mild *S. aureus)* are greater than 75% sensitivity and 93% specificity. PPV was greater than 80% and NPV was greater the 92%. Table 3 summarizes the sensitivity, specificity, negative predictive value, and positive predictive value.

### 3.5 Research question three

Lily, Bonny, and Bea were tested on each human target (*n* = 3 target samples) and biological control sample during one session (*n* = 8 control samples). Unfortunately, due to the COVID-19 pandemic, the testing of human samples was limited to one session on 17 March 2020 in which the three dogs were tested on the three target samples. Both Lily and Bonny gave final responses on patients B and C. Bea did not give a final response on any of the three target odors (Patients A, B or C) (Table 4). Patient A's sample was not correctly identified on first pass by any of the three dogs. The screening of further samples was not continued after 2020 due to withdrawal of the three detection dogs by their owners.

**Table 4:** Results of the three detection dogs for the *S. aureus* lacrimal fluid from human periprosthetic joint infection (PJI) patients target odor.

**Research Question 3:** Can the dogs involved in research questions 1 and 2 identify *S. aureus* in biosamples (lacrimal fluid) taken from humans with confirmed *S. aureus* PJI?								
	*S. aureus* lacrimal fluid Patients (*n* = 3)						Control lacrimal fluid patients (*n* = 8)	
	Patient A		Patient B		Patient C			
Dog	TP	FN	TP	FN	TP	FN	TN	FP
Lily	0	1	1	0	1	0	8	0
Bonny	0	1	1	0	1	0	7	1
Bea	0	1	0	1	0	1	7	1

## 4 Discussion

This study investigated the potential of training odor detection dogs using cultured *S. aureus* biofilms to detect biofilm-associated infections with a particular interest in the culture-negative patient. The results demonstrate that dogs trained on VOCs from cultured *S. aureus* biofilms display a high degree of sensitivity and specificity in recognizing *S. aureus* associated with PJI biofilm in lacrimal fluid from infected rabbits. Furthermore, because dogs trained on *in vitro* cultured *S. aureus* were able to generalize to biosamples obtained from a controlled rabbit model, it suggests that training dogs (or machines) on cultured *S. aureus* biofilms may provide an adequate VOC profile indicative of *S. aureus*, which may reduce logistical issues surrounding the acquisition of biological samples. Our preliminary results further suggest that this odor profile is consistent and appears to be detectable in human samples, however further testing is required. Our findings are interesting and suggest that there is a common VOC profile that is preserved across *S. aureus* biofilm from pure *in vitro* cultures and from lacrimal fluid biosamples obtained from rabbits with PJI secondary to *S. aureus* infection and human samples of naturally occurring *S. aureus* infection. This is intriguing since in the context of infections, the *S. aureus* VOC metabolome reflects complex host-microbial interactions throughout the processes of infection, yet this subset of metabolites detectable by the canine nose seems to be conserved. Moreover, our observations with odor detection dogs in the present study strongly support the potential of metabolomics-based diagnostics that can capture consistent metabolites for infection diagnosis, characterization, and monitoring (41). Diagnostic breath analysis is an area of intense research and its recent rapid advances have provided critical insight in how VOCs can differentiate closely related pathogens, including the capacity to establish antimicrobial sensitivity profiles (41). We elected lacrimal fluid as our source for biosamples because tear fluid contains high concentrations of proteins, is recognized as a potential source of biomarkers for other systemic disorders and collecting tear fluid via Schirmer strips is current standard of care when diagnosing dry eye disease (42). In addition, blood draws are more invasive, it carries VOC metabolites from all tissues and organs systems to the lungs and into exhaled breath potentially expanding the complexity of the VOC profile.

Our results for sensitivity and specificity in the detection of a consistent odor profile emanating from *S. aureus* biofilm infections align with previous studies that specialty trained odor detection dogs can differentiate between a target odor and controls. Although, in our study, the dogs' responses to the biosamples from confirmed *S. aureus* human patients were less consistent compared to the responses from the infected PJI rabbit samples. An explanation could be that these samples came from a new environment and the human lacrimal sample presented as a new target for the dogs. It is possible that, with additional testing of samples, dogs would have been able to recognize this odor with improving sensitivity and specificity. Many studies of odor detection dogs reveal an impressive sensitivity and specificity in detecting various VOC profiles emanating from several cancers and viruses in biologic samples (22, 30, 35, 39). These studies focused on several different sample types for disease detection in which negative control fluid samples were compared to positive clinical patients of the same substrate type (blood plasma vs. blood plasma from diseasepositive patients) (34) or less invasive samples were compared to more invasive samples [saliva vs. sweat and urine vs. saliva (43)]. Although results from these studies are encouraging, the reliance on samples from patients carrying the disease combined with appropriate controls for training of odor detection dogs may pose limitations in terms of sample acquisition, sample diversity, and generalization. This gap in accessibility dampens the commercial enthusiasm of this diagnostic approach, creating a major barrier in implementing odor detection dogs in a clinical diagnostic setting (26).

To address this gap, we proposed an alternative approach of training odor detection dogs on the VOC profile of the pathogen itself, such as cultured pure *in vitro S. aureus* biofilms using clinical strains. Training odor detection dogs on cultured samples may eliminate many challenges such as sample acquisition as they can be readily produced in the laboratory. In addition, training odor detection dogs on pathogen specific VOCs rather than on the pathogen itself, mitigates potential biosecurity hazards, and the need for biosafety measures during odor detection training. Recent studies have implemented this approach with different strains of the same bacterial and viral species (30, 31). Koivusalo et al. discovered odor detection dogs can detect and differentiate MSSA and Methicillin Resistant *Staphylococcus aureus* (MRSA) respectively with 97% sensitivity and 92% specificity (31). Angle et al. successfully trained dogs to differentiate between cultured cells infected with Bovine Viral Diarrheal Virus (BVDV) with a sensitivity of 85% and specificity of 98% (30). The high yield in sensitivity and specificity most likely is a result of sample variation and increased repetition during training amongst appropriate background odors, and further emphasizes the robustness of the canine odor detection test system.

The present study demonstrated encouraging results when utilizing cultured infectious disease pathogens such as *S. aureus* with a consistent target odor profile for training in preparation for diagnosing clinical samples. However, it is important to recognize that not all diseases lend themselves to *in vitro* culturing of a specific pathogen for training purpose of odor detection dogs. Murarka et al. explored the utility of odor detection dogs on cultured cell lines of ovarian cancer in hopes it would translate to blood plasma samples from clinical patients (38). Unfortunately, in that study only one dog could differentiate between the cultured ovarian cancer cell line and did not alert on the blood plasma samples when tested on clinical patients with confirmed late-stage ovarian cancer. The conflicting findings and experience with odor detection dogs in the field of cancer and infectious disease emphasize the importance of the underlying pathophysiology of the disease. The hallmark of an infectious disease processes is characterized by a pathogen invading a host triggering a host-microbial interaction. This process affords the training of odor detection dogs on the VOC profiles using pure cultures of a given pathogen. Conversely, the literature is inconclusive for disease processes which originate within the host such as cancer and whether the complexity of the hosts' environment, genetics, and metabolism on a given VOC profile, results in an inconsistent or convolute biomarker signature (34, 35, 44, 45). Further investigation into the interactions between the disease process, host, and source for training is warranted. Indeed, the ongoing controversy in the literature over the lack of well-defined standards and the clinical utility of odor detection dogs in the field of cancer and infectious diseases is perhaps a testament of the intensity of current research over the past 20 years and its considerable progress disentangling the chemistry on olfactory signatures of disease using dogs, advanced VOC analysis and clinical detection.

Our study did not evaluate polymicrobial biofilms, consisting of multiple bacterial species, which may pose challenges in training dogs on cultured biofilms that are composed of one microbial species. Conversely, lacrimal biosamples from non-human animals and human patients as it was the case in our studies are from subjects constantly challenged by microbial attacks. Furthermore, the human patients presenting for revision surgery in our study all underwent different antimicrobial regimes in attempt to treat the underlying clinical problem (PJI or FRI). Thus, while the VOC profiles of biofilm-associated infections may still vary among individuals and species, in our case of known *S. aureus* biofilm infection, dogs detected a seemingly consistent *S. aureus* specific odor profile. Undoubtedly, further research is needed to investigate the accuracy and generalizability of odor detection in dogs trained on cultured biofilms for detecting biofilm associated infections from host-derived biological samples. This could involve training dogs on biofilms from different species and strains, as well as comparing the performance of dogs trained on cultured biofilms with those trained on samples from infected individuals and appropriate controls. Additionally, studies comparing the VOC profiles of cultured biofilms with biological samples from infected individuals can provide insights into the similarities and differences in odor profiles and help determine the extent to which cultured biofilms represent the odor profiles of biofilm-associated infections. In fact, the findings by Kuil et al. further amplify the prospect toward differentiating closely-related pathogens of the same genus and species, perhaps even determining antimicrobial resistance and sensitivity based on unique VOC profiles associated with infection (46).

It should also be noted that because this initial study focused on the research question of whether dogs can generalize from *S. aureus in vitro* cultures to experimental infections in rabbits and naturally occurring PJI infections in human biosamples, we did not include control samples that represented other bacterial infections, or samples taken from people who were unwell with conditions other than *S. aureus*. Further studies should focus on specifically testing dogs' abilities to discriminate between (1) different types of biofilm infections, and (2) biosamples derived from patients who have confirmed PJI of *S. aureus* vs. samples taken from patients who are unwell with conditions other than a bacterial infection. This is important as it has been demonstrated in certain disease conditions, that several metabolic changes may occur, impacting an individual's VOC profile, when they become unwell (42). When considering other diseases and infections, previous studies have demonstrated dogs' abilities to specifically detect target samples of interest while ignoring similar conditions or samples indicative of “ill-health” unrelated to the target condition (36, 47, 48). These findings are well aligned with our observations. Our dogs have been trained on *in vitro* biofilms of *S. aureus* and continued to detect the target samples in experimental conditions of *S. aureus* biofilm in rabbits and in naturally occurring PJI of human patients undergoing various antimicrobial therapies, despite significant host-microbial interactions. This is important, since it is well accepted that *in vitro* and *in vivo* biofilms are not equivalent and host-microbial interactions significantly change biofilms. Clinical biofilm infections typically evolve with the patient undergoing antimicrobial therapy. He et al. recently demonstrated that quorum cheater emergence can be triggered during *S. aureus* biofilm infections using certain antimicrobials resulting in a significant increase of the bacterial burden and most importantly, the development of agr mutants (49).

Results of this study inform future developments in the field of culture-independent biofilm diagnostics and treatment. We have shown that not only does *S. aureus* have a detectable odor profile, but we also demonstrated that *in vitro* cultured *S. aureus* can be a viable training aid to be used to imprint dogs on *S. aureus*. The encouraging findings of odor detection dogs on infection diagnostics (50) combined with high cost and time-consuming training of dogs has inspired chemical analysts and engineers to emulate the dogs' capabilities of analyzing VOCs in biosamples. To this end, various studies have sought to build an electronic nose (28, 51, 52, 55, 56). The goal of an electronic nose is to mimic a bio-detection dog by analyzing VOCs, for example, by using metal-oxide sensors and learning algorithms. When exposed to a breath, blood, or urine sample, it probes a profile or so-called “smell print”. If proven effective, it would be a point-of-care, low-cost, handheld, noninvasive tool that overcomes many of the shortcomings associated with currently available methods. For now, dogs seem to outperform e-noses (53, 54, 57). Research needs to continue to pursue the elite abilities of odor detection dogs to investigate disease detection whilst simultaneously harnessing their skills to refine and enhance the e-nose technology for scalability to bring cultureindependent point-of-care-assays to the clinic floor.

## 5 Conclusion

Herein we demonstrated that dogs can be trained to detect a consistent odor profile associated with *in vitro* and *in vivo S. aureus* biofilms. Research questions one and two were determined to be successful in that dogs trained on *in vitro S. aureus* were able to generalize to *in vivo* lacrimal fluid from a non-human animal model (rabbits) at both a mild and moderate infection loads of *S. aureus* biofilms. Our findings are suggestive that host-microbial interactions do not alter certain VOC profile characteristics of *S. aureus* biofilm. Results discovered in research question three, further suggests that dogs could also generalize to *S. aureus* VOCs in human lacrimal fluid in patients presenting for orthopedic revision surgery. Taken together, these findings provide insight into *S. aureus* biofilm production as it relates to detectable VOC biomarkers that are conserved both *in vitro* and *in vivo* despite host-microbial interactions in experimental models of infection (rabbits) and naturally occurring biofilm infection in human patients. These findings may be critical in the design of continued research and development efforts into non-invasive, culture independent diagnostics for early disease detection, refined antimicrobial treatment, and improved antimicrobial stewardship.

## Data Availability

The raw data supporting the conclusions of this article will be made available by the authors, without undue reservation.
